# Prevalence of Dental Anomalies in Pediatric Patients at King Saud University Dental Hospital, Riyadh, Saudi Arabia—A Radiographic Analysis

**DOI:** 10.3390/children12010013

**Published:** 2024-12-25

**Authors:** Mannaa K. Aldowsari, Ayman M. Sulimany, Abdulmajeed Alkhathlan, Nawaf Alfhaed, Mohammed Aldosari, Saud Alayed, Saad Bin Saleh, Abeer A. Alshami

**Affiliations:** 1 Department of Pediatric Dentistry and Orthodontics, College of Dentistry, King Saud University, Riyadh 12372, Saudi Arabia; 2 College of Dentistry, King Saud University, Riyadh 12372, Saudi Arabia; 3 Department of Preventive Dental Sciences, College of Dentistry, Princess Nourah bint Abdulrahman University, P.O. Box 84428, Riyadh 11671, Saudi Arabia

**Keywords:** children, dental anomalies, hypodontia, ectopic eruption, prevalence

## Abstract

Objectives: To record the prevalence of dental anomalies in children visiting King Saud Dental Hospital, Riyadh, Saudi Arabia. Materials and Methods: This cross-sectional study involved radiographic examination of children aged 6 to 14 years who visited King Saud Dental Hospital, Riyadh, Saudi Arabia, in the past five years. Four dental interns were trained in three consecutive sessions by a pediatric dentist and evaluated the orthopantomograms of the children. The recorded anomalies were divided into four categories: tooth number, size, position, and general. Descriptive statistics were derived and chi-square tests performed to report prevalence and significance among genders, medical histories, and dental anomalies. The significance level was set at *p* < 0.05. Results: Of the 1987 radiographs studied, 268 (13.48%) children had dental anomalies. The sample consisted of 51.1% female and 48.5% male children, with a mean age of 11.87 ± 2.1 years. Most study participants had mixed dentition and were healthy. The prevalence of dental anomalies reported in this study was as follows: 4.6% hypodontia, 2.7% ectopic eruption, 1.63% taurodontism, 1.2% infra-occluded molars, 1.1% impacted teeth, 0.75% root dilaceration, and 0.65% supernumerary teeth. Significant correlations were found between gender and ectopic eruption (*p* = 0.02) and between medical history and hypodontia (*p* = 0.00), ectopic eruption (*p* = 0.048), and root dilaceration (*p* = 0.03). Conclusions: This study showed that hypodontia was the most common dental anomaly, followed by ectopic eruption and taurodontism. The findings of this study may guide dentists in better understanding, diagnosing, and treating dental anomalies in children in Riyadh, Saudi Arabia.

## 1. Introduction

Dental anomalies are deviations from the teeth’s standard form, number, size, structure, or color. These anomalies often occur during developmental stages and can be influenced by genetic, environmental, or combined factors [1]. Dental anomalies include morphological discrepancies, such as fusion, peg-shaped lateral incisors, and gemination; congenital anomalies, which primarily involve variations in tooth number, such as missing or supernumerary teeth; and anomalies related to tooth position, like ectopic eruption and rotation [2,3]. The etiology of dental anomalies is complex and multifactorial, often arising from congenital, acquired, and/or developmental factors [4].

The most common anomalies include changes in tooth number, such as hypodontia, the congenital absence of one or more teeth, and hyperdontia, where an extra tooth (supernumerary teeth) or teeth are present [5]. Tooth size anomalies, like macrodontia (enlarged teeth) and microdontia (reduced tooth size) can also occur [5]. Morphological anomalies, including fusion (where two tooth germs unite to form one large tooth), gemination (a partial division of a single tooth germ), and dens invaginatus (tooth within the tooth), further complicate dental function and aesthetics [1]. Structural anomalies, such as amelogenesis imperfecta and dentinogenesis imperfecta, affect enamel and dentin formation, leading to weak, discolored teeth that are more prone to caries [5,6]. Anomalies in tooth position, such as ectopic eruption and rotation, often impact alignment and may necessitate orthodontic intervention [6,7]. Additionally, dental anomalies are not limited to a single dentition; studies show that certain anomalies present in primary teeth can persist in permanent dentition [5,8].

Dental anomalies in the mandible and maxilla can lead to considerable aesthetic and functional challenges, ultimately affecting quality of life [9]. Additionally, studies indicate that dental anomalies present in primary dentition may also persist in permanent dentition. Global studies on the prevalence of dental anomalies have reported higher rates in children, potentially due to ethnic differences, variations in sampling methods, and differing diagnostic criteria [1,4,5,6,9,10,11]. However, a few global studies have reported similar prevalence rates of dental anomalies in children and adults [2,12,13,14].

Numerous studies have documented the prevalence of various dental anomalies, including hypodontia, microdontia, congenitally missing teeth, supernumerary teeth, gemination, fusion, and morphological defects [6,15,16,17,18]. A study in Italy involving 4706 children reported the highest prevalence of displaced maxillary canines (7.5%), followed by hypodontia (7.1%) [18]. In a cross-sectional survey conducted in Slough, England, gemination had a relatively higher prevalence (1.6%) than other anomalies, with no significant gender association observed [19]. In a comparative study on hypodontia prevalence, a significant difference was found between ethnic groups: Caucasian children had an incidence rate of 0.9% to 1.5%, while Swedish children had an incidence of 0.2% to 0.4% [20]. Additionally, Esenlik et al. examined 2599 radiographs of Turkish children and found a higher prevalence of supernumerary teeth (2.7%) [13].

A retrospective study in the eastern region of Saudi Arabia observed that the prevalence of teeth rotation was 24.5%, while that of ectopic eruption was 6.6% [6]. A cross-sectional study conducted in southern Saudi Arabia revealed that 2.2% of children had congenitally missing teeth, while supernumerary teeth and peg-shaped lateral incisors were present in 0.5% and 0.37% of cases, respectively [21]. According to Osuji and Hardie, children in Tabuk, Saudi Arabia, have a 3.6% prevalence of congenitally missing teeth [22]. Alfy and Zawawi reported a prevalence of congenitally missing teeth at 25.7% among adults in the western region of Saudi Arabia [9]. In 2016, Yassin conducted a study in the southwestern region of Saudi Arabia, revealing that approximately 9.7% of children exhibited congenitally missing teeth [21]. Dental anomalies have been documented in primarily adult populations in various regions of Saudi Arabia [23]. 

Multidisciplinary and complex approaches are required to treat dental anomalies in children. Hence, it is important to understand the prevalence of dental anomalies during the early stages. This approach will eventually help dentists and pediatric dentists to plan proper treatment and improve the quality of life of children. Nonetheless, evidence regarding the prevalence of dental anomalies among children in the Riyadh region of Saudi Arabia remains inadequate. Therefore, the present study aims to determine the prevalence of dental anomalies among children attending King Saud Dental Hospital in Riyadh, Saudi Arabia, through the analysis of dental radiographic records. This study further examines the relationship between dental anomalies and factors such as gender and medical history.

## 2. Materials and Methods

### 2.1. Study Design and Sample Selection

This study utilized a cross-sectional design, conducting a radiographic observational survey based on dental panoramic radiographs of pediatric patients who visited the Dental University Hospital at King Saud University in Riyadh, Saudi Arabia, between January 2014 and January 2023. The main reasons for patient visits to our hospital are pain, caries, malocclusion, and other dental anomalies; the consent of the patient’s legal guardians was obtained prior to any dental treatments, including dental x-rays. Ethical approval was obtained from the Institutional Review Board of King Saud University and Medical City (E-23-7973), and the study complied with the principles outlined in the Declaration of Helsinki. In accordance with the policy of King Saud University Dental Hospital, all patients were required to give their agreement prior to the opening of their files for their data to be utilized for educational and research reasons without disclosing their identities. The study followed STROBE guidelines. 

Statistical advice was obtained, and the sample size was calculated using the G-power sample size calculator (https://www.gigacalculator.com/calculators/power-sample-size-calculator.php) (accessed on 12 February 2023), with the following formula:

$$n_{1}=\frac{(Z_{1-\alpha }+Z_{1-\beta }{)}^{2}\times \sigma^{2}}{\delta^{2}}$$

where *n* is the sample size, *Z*_1−*α*_ denotes the Z-score associated with the chosen level of statistical significance (α), while *Z*_1−_*_β_* represents the Z-score related to the desired statistical power (1 − *β*). The standard deviation (σ) can be estimated analytically for proportions and derived empirically from raw data for other mean values, while *δ* indicates the minimum effect size (less than 5%) of interest. According to these data, a sample size of 1800 children was required for this study.

The inclusion criteria for the current study were as follows: the patient (1) should be in the mixed and/or permanent dentition phase, (2) must be aged between 6 and 14 years, and (3) must have undergone digital orthopantomography (OPG). Exclusion criteria included a history of head trauma, tooth loss due to trauma, missing teeth from extractions, low-quality radiographs, children above 14 years, and the presence of congenital anomalies. The Information Technology department at Dental University Hospital provided a spreadsheet (Microsoft Excel software 2010) of the patients’ case numbers who met the study inclusion criteria. Simple randomization was conducted, and radiographs were anonymized using serial numbers. Initial digital OPGs were reviewed, along with patient charts, to confirm the absence of any history of tooth extraction. Identifiable patient information, such as names and IDs, was not recorded, ensuring confidentiality.

### 2.2. Training and Calibration

Training and calibration of four dental interns were performed by a consultant pediatric dentist before conducting the study. The training took place in 3 consecutive sessions in the form of lectures, clinical training, and workshops. Dentists were taught to diagnose radiographs for different dental anomalies. Intra- and inter-examiner reliability was evaluated using a distinct set of radiographs separate from the test sample. Additionally, reassessment was conducted after a two-week interval, employing the kappa test as part of a pilot study. The answers of the included dentist were compared to those of an experienced pediatric dentist. The agreement was substantial (kappa ≥ 0.8).

### 2.3. Orthopantomogram (OPG) Assessment

Radiographs were obtained from an X-ray machine (PLANMECA—ProMax, Helsinki, Finland) in a designated radiographic reporting room with dimmed lighting. Four trained dental interns evaluated the images on a graphic monitor using the Planmeca Romexis dental radiographic software 6.0 (Helsinki, Finland). Simple image manipulation techniques, such as adjustments to contrast, brightness, and sharpness, were employed to enhance the accuracy of image interpretation. Data collection was conducted using a customized data collection sheet. Demographic information, including gender, age, and medical history, was recorded for each patient based on the available information in their files. A significant medical history was defined as the presence of any medical condition documented in the patient’s file, including details about medications.

All the anomalies identified on the radiographs were documented in a Microsoft Excel worksheet. The identified anomalies were systematically categorized into four distinct groups: anomalies pertaining to tooth number, which include supernumerary teeth and congenitally missing teeth; anomalies concerning tooth shape, encompassing macrodontia, microdontia, concrescence, gemination, fusion, taurodontism, regional odontodysplasia, dens evaginatus, and dens invaginatus; anomalies associated with tooth position, such as impaction, dilaceration, and tooth transposition; and general dental anomalies, which include taurodontism, amelogenesis imperfecta, and dentogenesis imperfecta (Table 1).

### Statistical Analysis

The Statistical Package for the Social Sciences (SPSS) version 25.0.0.2 (IBM Corp., Armonk, NY, USA) was used to analyze the data at a 5% significance level. Frequencies and percentages were calculated to record the prevalence, location, and quantity of each anomaly per patient. The chi-square test and Fisher’s exact test were employed to evaluate the differences in the prevalence of each anomaly in relation to the patient's gender and medical history. The significance level was established at 0.05. 

## 3. Results

A total of 1999 records were reviewed, of which 12 were excluded as they did not meet the study criteria. Finally, 1987 records were included in the current analysis. Table 1 represents the demographics of the included participants, where 48.9% (*n* = 972) were males and 51.1% *(n* = 1051) were females, with a mean age of 11.87 ± 2.10, respectively. Fifteen participants (0.75%) of 1987 participants had some chronic illness (Table 2).

Table 3 presents the prevalence of dental anomalies in children attending King Saud Dental Hospital. A total of 268 participants (13.48%) out of 1987 participants exhibited some dental anomaly. 

**Figure 1:**
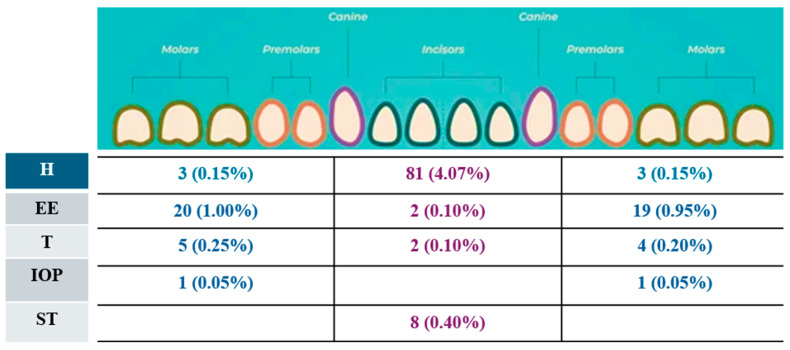
Number and prevalence of dental anomalies found in maxillary arch of affected children. H: hypodontia; EE: ectopic eruption; T: taurodontism; IOP: infra-occluded primary molars; ST: supernumerary teeth.

**Figure 2:**
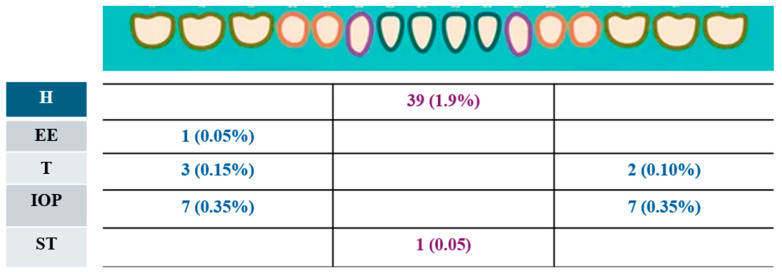
Number and prevalence of dental anomalies found in mandibular arch of affected children. H: hypodontia; EE: ectopic eruption; T: taurodontism; IOP: infra-occluded primary molars; ST: supernumerary teeth.

Two hundred sixty-eight participants (13.48%) exhibited some dental anomaly. The prevalence of hypodontia was 4.6% (*n* = 98) and located mainly in the upper and lower anterior (Figure 1 and Figure 2). The prevalence of other anomalies, included ectopic eruption, was observed in around 55 participants (2.7%), with one tooth involved in 42 cases (1.3%) (Figure 1 and Figure 2). This was followed by taurodontism in 33 participants (1.63%), infra-occluded molars in 24 participants (1.2%), impacted teeth in 23 participants (1.1%), root dilaceration in 15 participants (0.75%), and supernumerary teeth in 12 participants (0.60%) (Figure 1 and Figure 2). Fusion and macrodontia affected two participants (0.10%), while microdontia, ankylosis, dens evaginatus, and dens invaginatus were each observed in one participant (0.05%).

Table 4 illustrates the association between dental anomalies and both gender and medical history in children at King Saud Dental Hospital.

Gender-Based Analysis

Hypodontia was found to be evenly distributed between males (46.93%) and females (53.06%), with no significant difference (*p* = 0.570). The ectopic eruption was significantly more common in males (63.6%) than females (36.4%), with a *p*-value of 0.02. Other anomalies, including supernumerary teeth, macrodontia, and taurodontism, showed differences between genders; however, no significant association was seen (*p* > 0.05).

Medical History-Based Analysis

Hypodontia was significantly associated with medical history, occurring more frequently in children with documented medical conditions (*p* = 0.000) (Table 4). Ectopic eruption and root dilaceration were also more common in those with a medical history (*p* = 0.042 and *p* = 0.003). Other anomalies, such as supernumerary teeth, macrodontia, and taurodontism, did not significantly affect medical history (*p* > 0.05).

## 4. Discussion

This cross-sectional radiographic analysis investigated the prevalence of dental anomalies among children visiting King Saud Dental Hospital in Riyadh, Saudi Arabia. This study also assessed the association between these anomalies, gender, and medical history. Results indicated that approximately 13.48% (*n* = 268) of children exhibited some form of dental anomaly, with the highest prevalence being dental hypodontia (4.6%), particularly affecting the upper and lower anterior teeth (*n* = 81). The literature indicates that hypodontia is one of the most common dental anomalies, often resulting in aesthetic and functional complications that require multidisciplinary treatment. Previous studies in Saudi Arabia have reported the prevalence of congenitally missing teeth in 2.4% to 10% of children [9,10,21]. Celikoglu et al. found that 6.5% of Turkish children had hypodontia, primarily affecting maxillary laterals and premolars [24]. Similarly, Shokri et al. reported congenitally missing maxillary lateral incisors (12.3%), followed by premolars, in individuals aged 7–25 [25]. In a study by Goncalves-Filho et al., 11.12% of children aged 7–12 years had congenitally missed maxillary lateral incisors [11]. Similarly, Bakhurji et al. reported that 5.4% of children in the Eastern Province of Saudi Arabia were affected by hypodontia [1].

The etiology of ectopic eruption is not well documented but is thought to primarily involve genetic and local factors. Local factors such as a small arch, limited upper and lower jaw growth, early eruption of the maxillary first molar, bone growth at the tuberosity, and abnormal crown morphologies contribute to ectopic eruption [5,26]. In the present study, ectopic eruption was the second most common anomaly observed, with a prevalence of 2.7%. A significant association was found between ectopic eruption and gender (*p* = 0.02), with males (*n* = 35) being more affected than females (*n* = 20). This finding aligns with local and international studies. For example, a study in the southern region of Saudi Arabia reported a 7.6% prevalence of ectopic eruption, making it the second most common dental anomaly [20]. Similarly, a study from the Indian subcontinent reported a 7.6% prevalence of ectopic eruption and found a significant association with gender [2]. Conversely, Alfy and Zawawi observed ectopic eruption in only two children (0.3%) at King Abdulaziz Dental Hospital and found no significant association with gender [9].

The third most common anomaly reported in the current study is taurodontism (1.66%), predominantly affecting maxillary molars. However, studies on the prevalence of taurodontism are limited, with the most recent study on children in Trinidad and Tobago reporting a prevalence rate of 4.79% [27]. This rate is significantly higher than that found in the present study, possibly due to differences in sample size, ethnicity, and diagnostic criteria. Impacted teeth (*n* = 23) were also prevalent among the children analyzed in the current study. Impaction is one of the most common dental anomalies associated with tooth position. In a study by Laganà et al., the prevalence of impacted teeth was approximately 2.8%, and an association was found between impacted and supernumerary teeth [18]. The higher prevalence of impacted teeth observed in the current study may be due to the large number of cases among children aged 6 to 10. It is possible that some of these cases will resolve naturally over time and would not generally be classified as impacted teeth in later years.

The emergence of extra teeth, referred to as supernumerary teeth, may lead to various complications including crowding, delayed eruption, dental impaction, diastema, and the formation of cystic lesions [28,29]. The current study reported a low prevalence of supernumerary teeth. Further investigations carried out in different areas of Saudi Arabia revealed a low prevalence of supernumerary teeth among children, ranging from 0.50% to 3.5% [6,21]. Furthermore, Bäckman and Wahlin [15] documented a low prevalence of supernumerary teeth among Swedish children (1.9%), while Lagana et al. [18] observed a prevalence of 0.9% in Italian children. Kumar et al. [28] reported 0.96% in Turkish children, and Pallikaraki et al. [3] found a prevalence of 1% in Greek children. The significant variations in the distribution of dental anomalies reported in the literature can be ascribed to factors including sample size, age of study participants, and diagnostic criteria, in addition to genetic and racial influences.

The current study found that around 0.75% of children were affected by root dilaceration. This finding is like that in previous studies, where prevalence was reported to be between 0.70 and 5.8% in children with permanent dentition, mainly affecting maxillary incisors [30,31,32]. The literature suggests this condition occurs significantly more in the maxillary arch than the mandibular arch, with higher incidences in females [30,31,32]. The least common dental anomalies reported in the current study were macrodontia (0.1%), microdontia (0.04%), ankylosis (0.05%), and tooth transposition (0.05%). The prevalence of these anomalies varies by geographic, ethnic, and genetic factors. For instance, studies have reported that only 0.08% of Saudi children have macrodontia or microdontia [10,23]. Similarly, ankylosis is reported in only 0.05% of children in India [2]. Regarding tooth transposition, a few studies have shown that 0.03% of children are affected by this anomaly [33,34]. 

The current study reported a significant association between medical history and dental anomalies such as hypodontia, ectopic eruption, and root dilaceration. Similarly, a previous study on Swedish children found a significant association between medical history and ectopic eruption [18]. In the study by Bakhurji et al., a significant association was also found between medical history and various dental anomalies among Saudi children [1]. Conversely, a Turkish study reported no significant association between medical history and dental anomalies [35].

This is the first study to report the prevalence and distribution of dental anomalies among the children visiting King Saud Dental Hospital. It is important to understand dental anomalies in this hospital, as the outpatient unit is the most frequently attended. Moreover, this study will provide insight into proper diagnostic measures and appropriate treatment. For instance, in this study, hypodontia is reported as the highest among all the anomalies. This information will be helpful to dentists and pediatric dentists who observe this specific anomaly while examining children, and help prepare these dentists to manage it. Data accuracy was given importance in this study by training and calibrating the dentists, with excellent inter- and intra-examiner reliability. 

Certain limitations to this study should be considered when interpreting the results. Firstly, ethnic background and nationality were not considered because this information was unavailable in the patients' medical histories. Secondly, the sample size was limited to patients attending King Saud Hospital, restricting the ability to generalize the findings. Future research should focus on nationally representative samples to investigate dental anomalies among children in Saudi Arabia.

## 5. Conclusions

The results of this study showed that around 13.48% of participants had some type of anomaly. Among the dental anomalies studied, hypodontia was reported most often, followed by ectopic eruption, taurodontism, impacted teeth, and root dilaceration. In contrast, anomalies such as fusion, dens evaginates, ankylosis, and tooth transposition were the least common. A significant association was found between ectopic eruption and gender, while hypodontia, ectopic eruption, and root dilaceration were significantly associated with medical history. These findings may serve as a guideline for dental practitioners and pediatric dentists to improve the diagnosis and management of dental anomalies in children in Riyadh, Saudi Arabia.

## Figures and Tables

**Table 1:** Dental anomalies categorized in the current study [5,6,8,18].

Dental Anomaly	Characteristic Features	Radiographical Features
***Anomalies in tooth number***		
*Hypodontia*	Absence of one or more teeth, commonly affecting premolars and lateral incisors.	Absence of tooth buds in the expected positions in the dental arch at the expected dental age Increased spacing or gaps between teeth. Altered eruption patterns of adjacent teeth.
*Hyperdontia (Supernumerary Teeth)*	Presence of extra teeth beyond the normal number, often in maxillary incisors (mesiodens).	Additional tooth-like structures, often conical or tuberculate, in atypical locations (e.g., mesiodens between maxillary central incisors). Crowding or displacement of adjacent teeth.
***Anomalies in the shape***		
*Microdontia*	Abnormally small teeth, usually affecting maxillary lateral incisors (peg laterals).	Reduced dimensions of crowns and roots. Peg-shaped teeth, especially in maxillary lateral incisors (peg laterals)
*Macrodontia*	Unusually large, less common teeth can affect single or multiple teeth.	Increased crown and root dimensions relative to adjacent teeth. Possible crowding due to oversized teeth.
*Fusion*	Union of two tooth buds, resulting in a single large tooth with two roots or pulp chambers.	Bifrid crown or two tooth buds fused. Mainly seen in lateral incisors and molar tooth
*Gemination*	Partial division of a single tooth bud, leading to a large tooth with a single root	Enlarged crown with a single root and pulp chamber. Clear separation line on the crown, but the root system remains single.
*Dens Invaginatus*	Invagination of enamel into the dental papilla, creating a “tooth within a tooth” appearance.	Radiolucent invagination extending from the crown inward toward the pulp. Commonly seen in maxillary lateral incisors.
*Dens Evaginatus*	An extra cusp or tubercle is present, often on premolars in Asian populations.	Small, well-defined radiopaque projection on the occlusal surface of affected teeth. Potential pulp extension into the projection.
***General dental anomalies***		
*Taurodontism*	Enlarged pulp chamber with shortened roots, typically seen in molars.	Vertically elongated pulp chamber with shortened roots. Increased distance between the cemento-enamel junction (CEJ) and the pulpal floor.
*Amelogenesis Imperfecta*	Defective enamel formation leads to thin, rough, or pitted enamel surfaces.	radiographically absent enamel layer, depending on the type
*Dentinogenesis Imperfecta*	Opalescent, discolored teeth with weak dentin, prone to wear and fracture.	bulbous crowns, cervical constriction, short and slender roots, and obliterated pulp chambers.
*Enamel Hypoplasia*	Thin or pitted enamel is caused by disrupted enamel formation, often due to early childhood illness or trauma.	localized areas of reduced enamel thickness or density, which could appear as distinct lines or pits on the enamel surface.
*Turner’s Hypoplasia*	Localized enamel defect is usually caused by trauma or infection that affects the development of teeth.	Localized reduction in enamel thickness or density. May appear as irregular radiolucent defects in the crown enamel.
***Dental anomalies according to position***		
*Impaction*	They are commonly impacted, especially by the maxillary canines, due to lack of space or misalignment.	Tooth positioned below the alveolar bone level, often obstructed by another tooth, bone, or soft tissue. Frequently seen with third molars and canines.
*Transposition*	The position of the canine may be swapped with other teeth, often the lateral incisor or first premolar.	Clear angulation or curvature in the root or crown axis. Commonly caused by trauma to the developing tooth.
*Infra-occluded molars*	Primary molars failed to maintain its position in the dental arch and is below the occlusal plane of adjacent teeth, often due to ankylosis or delayed resorption.	The step between the infraoccluded tooth and the adjacents, the common bilateral presentation, and the presence of obliteration of the periodontal ligament
*Dilaceration*	An abnormal bend in the root, often due to trauma affecting tooth development.	Altered eruption pattern with teeth appearing in switched positions (e.g., canine and premolar). Roots may overlap or cross in panoramic images
*Ankylosis or failure of eruption of permanent molars*	An abnormal disorder that defind as incomplete tooth eruption of permentent molars despite the absence of obstructed eruption pathway	Tooth positioned below the alveolar bone level, despite the clear eruption pathway.

**Table 2:** Demographic distribution of study subjects (*n* = 1987).

Variables		*n* (100%)
Gender	Male	972 (48.9%)
	Female	1015 (51.1%)
Medical history	Yes	15 (0.75%)
	No	1972 (87.94%)
Age	Mean ± SD	11.87 ± 2.1056
	Range	6–14 years old

*n* = sample size.

**Table 3:** Prevalence and distribution of anomalies according to teeth location and teeth number among the affected children (*n* = 1987).

Anomalies Type	*n* (%)	Location					Number of Teeth Affected	
		Lower Anterior	Upper Anterior	Lower Posterior	Upper Posterior	Both	One	≥2
Hypodontia in perennate teeth, excluding third molars	98 (4.9%)	6 (0.39%)	6 (0.39)	0 (0.0%)	5 (0.30%)	81(4.0%)	17 (0.85%)	81 (4.0%)
Supernumerary teeth	12 (0.60%)	1 (0.05%)	8 (0.40%)	0 (0.0%)	0 (0.0%)	3 (0.10%)	9(0.45%)	3(0.10%)
Macrodontia	2 (0.10%)	0 (0.0%)	0 (0.0%)	0 (0.0%)	0 (0.0%)	2 (0.10%)	0 (0.0%)	2 (0.10%)
Microdontia	1 (0.05%)	0 (0.0%)	1 (0.05%)	0 (0.0%)	0 (0.0%)	0 (0.05%)	1 (0.05%)	0 (0.0%)
Fusion	2 (0.10%)	2 (0.10%)	0 (0.0%)	0 (0.0%)	0 (0.0%)	0 (0.0%)	2 (0.10%)	0 (0.0%)
Ankylosis or failure of eruption of permanent molars	1 (0.05%)	0 (0.0%)	0 (0.0%)	0 (0.0%)	0 (0.0%)	1 (0.05%)	0 (0.0%)	1 (0.05%)
Ectopic eruption	55 (2.7%)	0 (0.0%)	2 (0.10%)	1 (0.05%)	39 (1.9%)	13 (0.65%)	42 (2.1%)	13 (0.65%)
Impacted teeth	23 (1.1%)	0 (0.0%)	9 (0.49%)	1 (0.05%)	3 (0.29%)	10 (0.50%)	13 (0.65%)	10 (0.50%)
Tooth transposition	1 (0.05%)	0 (0.0%)	1 (0.05%)	0 (0.0%)	0 (0.0%)	0 (0.0%)	1 (0.05%)	0 (0.0%)
Root dilacerations	15 (0.75%)	2 (0.10%)	1 (0.05%)	5 (0.30%)	4 (0.20%)	3 (0.10%)	12 (0.60%)	3 (0.10%)
Taurodontism	33 (1.66%)	0 (0.0%)	2 (0.10%)	5 (0.30%)	9 (0.45%)	17 (0.85%)	16 (0.79%)	17 (0.85%)
Infra-occluded primary molars	24 (1.2%)	0 (0.00%)	0 (0,00%)	14 (0.45%)	2 (0.10%)	8 (0.40%)	16 (0.79%)	8 (0.40%)
Dens evaginates	1 (0.05%)	0 (0.0%)	1 (0.05%)	0 (0.0%)	0 (0.0%)	0 (0.0%)	1 (0.05%)	0 (0.0%)
Dens invaginatus	1 (0.05%)	0 (0.0%)	1 (0.05%)	0 (0.0%)	0 (0.0%)	0 (0.0%)	1 (0.05%)	0 (0.0%)

*n* = sample size.

**Table 4:** Distribution of dental anomalies by gender and medical history among the affected children (*n* = 268).

	**Gender**		
**Anomalies**	**M**	**F**	***p*** **-Value**
Hypodontia in perennate teeth excluding third molars	46 (46.93%)	52 (53.06%)	0.570
Supernumerary teeth	9 (75.0%)	3 (25.0%)	0.085
Macrodontia	1(50.0%)	1(50.0%)	1.000
Microdontia	1(100%)	0 (0.00%)	0.51
Fusion	0 (0.00%)	2 (100%)	0.500
Ankylosis or failure of eruption of permanent molars	1(100%)	0 (0.00%)	0.489
Ectopic eruption	35 (63.6%)	20 (36.4)	0.02 *
Impacted teeth	9 (39.1%)	14 (60.9%)	0.40
Tooth transposition	0 (0.00%)	1(100%)	1.000
Root dilacerations	4 (26.7%)	11(73.3%)	0.118
Taurodontism	22 (66.7%)	11 (33.3%)	0.052
Infra-occluded primary molars	10 (42.7%)	14 (58.33%)	0.541
Dens evaginates	0 (0.00%)	1(100%)	1.000
Dens invaginatus	0 (0.00%)	1 (100%)	1.000
	**Medical History**		
**Anomalies**	**Yes**	**No**	***p*** **-** **Value**
Hypodontia in perennate teeth excluding third molars	9	8	0.000 *
Supernumerary teeth	0	12	1.000
Macrodontia	0	2	1.000
Microdontia	0	1	1.000
Fusion	0	2	1.000
Ankylosis or failure of eruption of permanent molars	0	1	1.000
Ectopic eruption	2	52	0.042 *
Impacted teeth	1	22	0.131
Tooth transposition	0	1	1.000
Root dilacerations	2	13	0.003 *
Taurodontism	1	32	0.183
Infra-occluded primary molars	0	24	1.000
Dens evaginates	0	1	1.000
Dens invaginatus	0	1	1.000

M = male; F = female; *p*-value = 0.05 *.

## Data Availability

The original contributions presented in this study are included in the article. Further inquiries can be directed to the corresponding author.
